# Temporal Trends and Correlates of Physical Activity, Sedentary Behaviour, and Physical Fitness among School-Aged Children in Sub-Saharan Africa: A Systematic Review

**DOI:** 10.3390/ijerph110303327

**Published:** 2014-03-20

**Authors:** Stella K. Muthuri, Lucy-Joy M. Wachira, Allana G. Leblanc, Claire E. Francis, Margaret Sampson, Vincent O. Onywera, Mark S. Tremblay

**Affiliations:** 1 Children’s Hospital of Eastern Ontario Research Institute, 401 Smyth Road, Ottawa, ON K1H 8L1, Canada; E-Mails: alleblanc@cheo.on.ca (A.G.L.); cefrancis89@hotmail.com (C.E.F.); msampson@cheo.on.ca (M.S.); mtremblay@cheo.on.ca (M.S.T.); 2 Population Health, Faculty of Graduate and Postdoctoral Studies, University of Ottawa, 1 Stewart Street, Ottawa, ON K1N 7M9, Canada; 3 Department of Physical and Health Education, Kenyatta University, P.O. Box 43844, Nairobi, 00100, Kenya; E-Mail: mwlucyjoy@yahoo.com; 4 Department of Recreation Management and Exercise Science, Kenyatta University, P.O. Box 43844, Nairobi, 00100, Kenya; E-Mail: vonywera@gmail.com

**Keywords:** motor activity, sedentary lifestyle, physical fitness, child, adolescent, Sub-Saharan Africa

## Abstract

Recent physical activity (PA) and fitness transitions, identified as behavioural shifts from traditionally active lifestyles to more industralised and sedentary lifestyles, have been observed among school-aged children. There is a wealth of supporting evidence of such behavioural transitions in high income countries; however, a paucity of data on lower income countries exists. These transitions pose a particular threat to the welfare of children by accelerating the onset of chronic diseases. This systematic review investigated the evidence for a PA and fitness transition among Sub-Saharan Africa’s school-aged children. Temporal trends and correlates of PA, SB, and fitness were examined. Studies were identified by searching the MEDLINE, Embase, Africa Index Medicus, Global Health, Geobase, and EPPI-Centre electronic databases, and were included if they measured outcomes of interest in apparently healthy samples of children (5‒17 years). A total of 71 articles met the inclusion criteria (40 informed PA, 17 informed SB, and 37 informed fitness). Vast heterogeneity in study methodology complicated analysis of transitions over time and no temporal trends were immediately discernible. However, higher socioeconomic status, urban living, and female children were found to engage in lower levels of PA, higher SB, and performed worse on aerobic fitness measures compared to lower socioeconomic status, rural living, and male children. Data revealed that urbanization was associated with a trend towards decreased PA, increased SB, and decreased aerobic fitness over time. Representative, temporally sequenced data examining a PA and fitness transition are lacking in this region (PROSPERO Registration Number: CRD42013004399).

## 1. Introduction

Global surveillance efforts have revealed a behavioural shift from traditionally active lifestyles, to more industrialised and sedentary lifestyles [1]. The resultant decline in physical activity levels, coupled with increasing sedentary behaviours over time, is referred to as a “physical activity transition” [1]. The World Health Organization (WHO) classifies physical inactivity as the fourth leading cause of global mortality, and a major determinant for various chronic diseases [2]. Indeed, maintaining an adequate level of physical activity and reducing the amount of time spent in sedentary pursuits is important for the prevention of chronic disease morbidity and mortality [1]. Global physical activity transitions may pose a particular threat to the welfare of children and youth due to the possibility of long-term co-morbidities. 

While communicable diseases will likely remain the predominant health problem for the populations in Sub-Saharan Africa (SSA) in the coming years, there is growing concern about rapid increases in non-communicable diseases (NCDs) such as heart disease, diabetes, and hypertension, particularly in urban areas [3]. SSA is currently undergoing rapid socio-cultural developments and urbanization, which have led to the replacement of an economy based on manual labour, to one dominated by industry and mechanized manufacturing [4,5]. This has also resulted in shifts in habitual and occupational physical activity from high-energy expenditure activities (e.g., active transport, manual labour) to low-energy expenditure activities or sedentary behaviours (e.g., motorized transport, desk work) [1]. A transition to lower levels of physical activity is thought to be one of the main contributors to the increasing burden of preventable NCDs in SSA [1,4,6,7,8]. Of great concern, are the long-term health consequences on children and youth, who are also influenced by this physical activity transition.

A large scale study of secular trends of children and adolescents between 1980 and 2000 using data from 11 mainly developed countries around the world revealed that aerobic fitness (measured by shuttle run tests) had rapidly declined, with the most marked decrease occurring in older age groups, and the rate of decline was similar for boys and girls [9]. This study provided supporting evidence for a decline in physical fitness, particularly aerobic fitness, among children and youth in developed countries. 

Whereas physical activity and fitness transitions are well monitored and known to have taken place in high income countries such as Canada, the United States, or the United Kingdom; there is little information on possible physical activity and fitness transitions in SSA, particularly in the school-aged population. Indeed, SSA may be at the early stages of these transitions, and possibly amenable to early intervention strategies aimed at preserving healthy active behaviours. Therefore, the objective of this systematic review was to examine the evidence for a physical activity and fitness transition occurring among SSA’s school-aged children and youth. Specifically, temporal trends and correlates of physical activity, sedentary behaviour, and physical fitness in SSA countries were examined. It is important to note that these terms are not interchangeable, particularly among children and youth, as described in Table 1. 

**Table 1:** Outcome measures and their descriptions.

Outcome	Description
Physical Activity	Adequate participation in energy expending activities (e.g., walking, cycling, dancing) provides a wide spectrum of health benefits including reductions in risk for a variety of diseases, improvements in functional ability, and promotes psychological well-being [3,10,11]. Global physical activity guidelines recommend that children and youth, 5-17 years of age, should accumulate at least 60 min of moderate to vigorous intensity physical activity daily [12].
Sedentary Behaviours	These activities (e.g., prolonged sitting, seated screen time, motorized transportation) are characterized by sitting or reclined posture, little physical movement, and low energy expenditure (<1.5 metabolic equivalent tasks) [13]. Canadian sedentary behaviour guidelines developed by the Canadian Society for Exercise Physiology state that for health benefits, children aged 5–17 years should minimize the time they spend being sedentary each day by limiting recreational screen time to no more than 2 h per day, limiting motorized transport, extended sitting, and time spent indoors throughout the day [14].
Physical Fitness	Includes a set of health or skill related attributes that individuals possess in order to perform physical activity [15]. Health related components of physical fitness include cardiorespiratory endurance/aerobic (measured by cycle ergometer, shuttle run, distance run, Harvard step test *etc*.), musculoskeletal endurance & fitness (measured by pull ups, bent-arm hangs, sit ups *etc*.), muscular strength (measured by hand grip, trunk lift etc), body composition measures (measured by body mass index, body fat percentage, waist circumference *etc*.), flexibility (measured by sit-and-reach *etc*.), and anaerobic power (measured by dash/sprint runs, jumps *etc*.) [15].

## 2. Methods

### 2.1. Study Inclusion Criteria

All published, peer-reviewed studies were included if they reported using subjective or objective measures of physical activity, sedentary behaviour, or physical fitness in children and youth aged 5 to 17 years, with no chronic conditions, and living in SSA. The relevant outcome measures are described in Table 1.

### 2.2. Study Exclusion Criteria

In order to obtain information on a general population living under typical conditions, intervention studies were excluded unless they conducted baseline measurements. No date limits were imposed, but due to feasibility, studies in languages other than English or French were excluded. Studies were also excluded if they did not include one or more of the relevant health indicators.

### 2.3. Search Strategy

Studies were identified using the following electronic databases: Ovid MEDLINE (1948 to Week 4, May 2013), Ovid Embase (1974 to Week 21, 2013), Africa Index Medicus (database dates not available, searched on 3 June 2013), Global Health (1973 to 3 June 2013 through the CAB direct interface), Geobase (1884 to 3 June 2013 through the Engineering Village interface), and EPPI-Centre database of health promotion research (Bibliomap) (dates of coverage not available, searched 3 June 2013). The search strategy for this systematic review was completed in tandem with another publication examining the evidence for an overweight/obesity transition among school-aged children and youth in SSA [16]; hence, the inclusion of these terms in the search strategy. The search strategy was created and run by a research librarian. The complete search strategy used for MEDLINE is presented in Table 2. References were exported and de-duplicated using Reference Manager Software (Version 11, Thompson Reuters, San Francisco, CA, USA). Titles and abstracts of potentially relevant articles were screened by two independent reviewers, and full text articles were obtained for those meeting initial screening criteria. The full text articles were then screened in duplicate for inclusion in the review. This review is registered with the international prospective register of systematic reviews PROSPERO network (registration number: CRD42013004399) [17]. 

**Table 2:** MEDLINE search strategy; Ovid interface.

Order	Search Terms
1	exp “Africa South of the Sahara”/
2	(sub-sahar * or east afric * or south afric * or keny * or (south adj3 sahar *)).mp.
3	1 or 2
4	sedentar$.tw.
5	Sedentary Lifestyle/
6	((chair or sitting or car or automobile or auto or bus or indoor or in-door or screen or computer) adj time).tw.
7	low energy expenditure.tw.
8	(computer game * or video game * or ((television adj watch *) or tv watch *)).tw.
9	television/ or computers/ or video games/
10	(screen based entertainment or screen-based entertainment or screen time).tw.
11	physical inactivit *.tw.
12	bed rest.mp.
13	sitting.tw.
14	exp obesity/
15	(obesity * or obese).tw.
16	exp overweight/
17	(overweight or over weight).tw.
18	exp Body Fat Distribution/
19	exp body composition/
20	Waist Circumference/
21	waist circumference.tw.
22	Skinfold Thickness/
23	(skin folds or skin fold *).tw.
24	(body composition * or BMI or body mass index).tw.
25	exp “body weights and measures”/
26	(bio-impedance analysis or BIA).tw.
27	Absorptiometry, Photon/
28	(absorptiometery or densitometry or photodensitometry or DXA or DEXA).tw.
29	Physical Fitness
30	(physical conditioning or physical fitness).tw.
31	musculoskeletal fitness.tw.
32	physical endurance/
33	cardiovascular fitness.tw.
34	motor activit$.tw.
35	physical exertion/
36	aerobic exercise.tw.
37	exp sports
38	play/
39	exp physical education/
40	musculoskeletal physiological processes/ or exercise/ or movement/ or locomotion/ or running/ or swimming/ or walking/ or motor activity/
41	or/4–40
42	(child * or adolescent * or youth * or pediatric * or paediatric *).tw.
43	3 and 41 and 42

Notes: The search strategy for this systematic review was completed in tandem with a sister publication examining the evidence for an overweight/obesity transition among school-age children and youth in Sub Saharan Africa [16]; hence, the inclusion of these terms in the search strategy.

### 2.4. Data Extraction, Synthesis and Quality Assessment

Data extraction was completed using a standardized data extraction template, and study quality was assessed using a modified Downs and Black instrument [18]. The Downs and Black checklist for measuring quality of evidence was selected for use due to its suitability for quality assessment of original research articles (beyond the typical gauges used for quality assessment of evidence from systematic reviews and meta-analyses) [18]. Due to limitations in study design, questions selected from the Downs and Black quality assessment instrument excluded any questions that referred to intervention and trial study methodology, leaving ten out of a possible 27 questions, as represented in Table 3.

**Table 3:** Modified Downs and Black checklist [18].

**Reporting**
Objective Clearly Stated—Question 1 from full checklist (Y = 1/N = 0)
Main Outcomes Clearly Described—Question 2 (Y = 1/N = 0)
Patient Characteristics Clearly Defined—Question 3 (Y = 1/N = 0)
Main Findings Clearly Defined—Question 6 (Y = 1/N = 0)
Random Variability in Estimates Provided—Question 7 (Y = 1/N = 0)
Actual Probability Values Reported—Question 10 (Y = 1/N = 0)
**External Validity**
Sample Targeted Representative of Population—Question 11 (Y = 1/N = 0)
Sample Recruited Representative of Population—Question 12 (Y = 1/N = 0)
**Internal Validity/Bias**
Statistical Tests Used Appropriately—Question 18 (Y = 1/N = 0)
Primary Outcomes Valid/Reliable—Question 20 (Y = 1/N = 0)

## 3. Results

**Figure 1:**
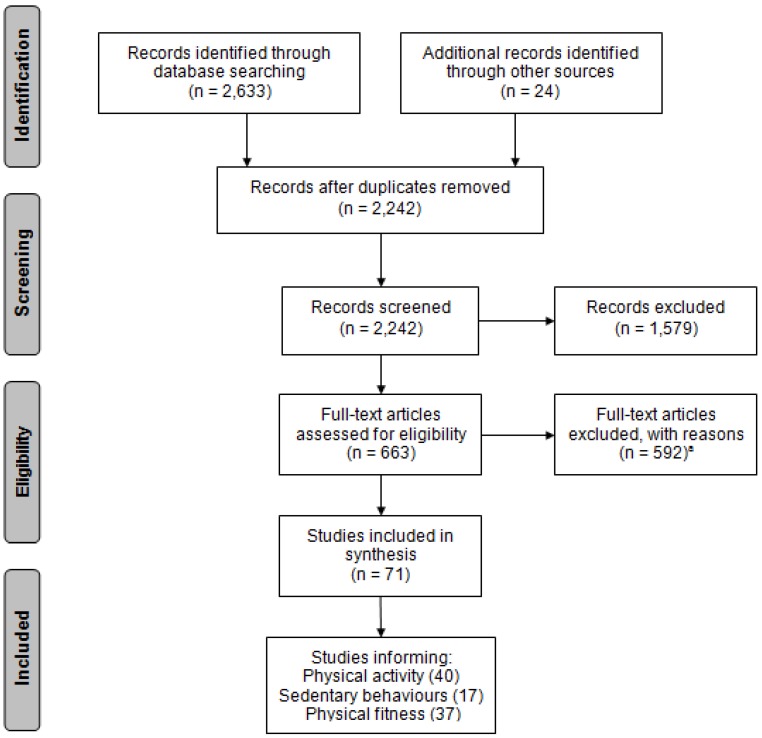
PRISMA flow chart of search strategy results.

**Notes:** PRISMA flow diagram [89]: **^a^** Reasons for exclusion included irrelevant population (181 studies), irrelevant geographic location/not Sub Saharan Africa (10 studies), irrelevant outcomes (334 studies), and irrelevant content/study design e.g. intervention programs or review articles (67 studies).

Figure 1 shows the PRISMA flow diagram with numbers of included and excluded articles at each step of the review process, while Table 4 provides a summary of all studies included in this systematic review. A total of 2,657 records were identified through database searches and other sources. Following duplicate removal, 2,242 were screened for eligibility, and 663 were selected for a full-text review. Of these, a total of 71 articles met inclusion criteria, comprising a total sample of 77,515 participants from 17 SSA countries. Reasons for exclusion included: irrelevant population (e.g., studies that did not involve children 5–17 years of age with no pre-existing condition) (181 articles); population living in a country outside of SSA (10); irrelevant outcomes (334); and, excluded study design (67 articles). 

**Table 4:** Descriptive characteristics of included studies.

First Author [reference]	Year					Study Design					Country			Age (Years)		Sample (n)	Outcome (Measures)				D&B Score	
Sloan [19]	1967					Cross sectional					South Africa			15–17		393	PF (anaerobic fitness, musculoskeletal fitness)				7	
Areskog [20]	1969					Cross sectional					Ethiopia			9–14		153	PF (aerobic fitness, musculoskeletal fitness)				7	
Stephenson [21]	1985					Cross sectional					Kenya			7–15		12	PF (aerobic fitness)				7	
Corlett [22]	1986					Cross sectional					Botswana			6–11		289	PF (musculoskeletal fitness)				6	
Ndamba [23]	1986					Cross sectional					Zimbabwe			8–15		147	PF (aerobic fitness)				7	
Corlett [24]	1988					Cross sectional					Botswana			7–12		612	PF (musculoskeletal fitness)				7	
Benefice [25]	1992					Cross sectional					Senegal			9–14		100	PF (aerobic fitness, anaerobic fitness, musculoskeletal fitness)				7	
Proctor [26]	1996					Cross sectional					Cameroon			9–14		119	PA (self-report)				7	
Benefice [27]	1996					Cross sectional					Senegal			5–13		348	PF (aerobic fitness, anaerobic fitness, musculoskeletal fitness)				7	
Benefice [28]	1998					Cross sectional					Senegal			5–13		348	PF (aerobic fitness, anaerobic fitness, musculoskeletal fitness)				7	
Prista [29]	1998					Cross sectional					Mozambique			8–15		593	PA (self-report) & PF (aerobic fitness, musculoskeletal fitness)				8
Benefice [30]	1999					Cross sectional					Senegal			12–13		221	PA (direct measures)				8
Benefice [31]	2001					Cross sectional					Senegal			13		40	PA (direct measures)				8
Benefice [32]	2001					Cross sectional					Senegal			13		40	PA (direct measures)				8
Garnier [33]	2001					Cross sectional					Senegal			13–15		80	PA (direct measures) & SB (direct measures)				8
Prista [34]	2003					Cross sectional					Mozambique			6–17		2,316	PA (self-report) & PF (musculoskeletal fitness)				8
McVeigh [35]	2004					Cross sectional					South Africa			9		386	PA (self-report) & SB (self- report)				7
McVeigh [36]	2004					Cross sectional					South Africa			10		386	PA (self-report)				7
Micklesfield [37]	2004					Cross sectional					South Africa			7–11		198	PA (self-report)				7
Larsen [38]	2004					Cross sectional					Kenya			15–17		11	PA (self-report)				7
Benefice [39]	2004					Cross sectional					Senegal			13–15		40	SB (direct measures)				7
Monyeki [40]	2004					Longitudinal					South Africa			7		85	PF (aerobic fitness, anaerobic fitness, musculoskeletal fitness, balance/flexibility)				8
Monyeki [41]	2005					Longitudinal					South Africa			7–14		855	PF (aerobic fitness, anaerobic fitness, musculoskeletal fitness)				8
Benefice [42]	2005					Cross sectional					Senegal			10–13		99	PA (direct)				8
Aandstad [43]	2006					Cross sectional					Tanzania			9–10		156	PA (self-report) & PF (aerobic fitness)				7
Garnier [44]	2006					Cross sectional					Senegal			13–15		80	SB (direct measures)				7
Djarova [45]	2006					Cross sectional					Zimbabwe			6–14		49	PA (self-report) & PF (musculoskeletal fitness)				6
Onyewadume [46]	2006					Cross sectional					Botswana			11–14		30	PF (musculoskeletal fitness)				8
Micklesfield [47]	2007					Cross sectional					South Africa			9		64	PA (self-report) & SB (self-report) & PF (musculoskeletal fitness)				7
Monyeki [48]	2007					Longitudinal					South Africa			7–14		702	PF (aerobic fitness, anaerobic fitness, musculoskeletal fitness)				8
Bovet [49] ^a,b,c^	2007					Cross sectional					Seychelles			12–15		4,343	PF (aerobic fitness, anaerobic fitness, musculoskeletal fitness)				9
Travill [50]	2007					Cross sectional					South Africa			8–17		720	PF (anaerobic fitness, musculoskeletal fitness, balance/flexibility)				7
Monyeki [51]	2008					Longitudinal					South Africa			7–13		1,817	PF (aerobic fitness, anaerobic fitness, musculoskeletal fitness, balance/flexibility)				7
Lennox [52]	2008					Cross sectional					South Africa			15		318	PA (self-report) & SB (self -report) & PF (musculoskeletal fitness, balance/flexibility)				8
Prista [53]	2009					Cross sectional					Mozambique			6–16		256	PA (direct measures)				8
Berntsen [54]	2009					Cross sectional					Tanzania			9–10		190	PF (aerobic fitness)				8
Peltzer [55]	2009					Secondary analysis					Namibia, Kenya, Uganda, Zimbabwe			13–15		12,740	PA (self-report) & SB (self- report)				9
Peltzer [56]	2010					Secondary analysis					Botswana, Kenya, Namibia, Senegal, Swaziland, Uganda, Zambia, and Zimbabwe			13–15		24,593	PA (self-report) & SB (self- report)				9
Harmse [57]	2010					Cross sectional					South Africa			13–17		221	PF (aerobic fitness)				7
Senbanjo [58]	2010					Cross sectional					Nigeria			5–14		392	PA (self-report)				8
Truter [59]	2010					Cross sectional					South Africa			9–13		280	PF (aerobic fitness)				7
Odunaiya [60]	2010					Cross sectional					Nigeria			14–16		608	PA (self-report)				7
Ansa [61]	2010					Cross sectional					Nigeria			10–17		964	PA (sel- report)				8
Adeniyi [62]	2011					Cross sectional					Nigeria			13–17		1,100	PA (self-report)				7
Peltzer [63] ^c^	2011					Secondary analysis					Ghana & Uganda			13–15		5,613	PA (self-report) & SB (self- report)				9
Naude [64]	2011					Cross sectional					South Africa			12–16		162	PA (self-report) & SB (self -report)				5
Croteau [65]	2011					Cross sectional					Kenya			8–12		72	PA (direct measures)				8
Muller [66]	2011					Cross sectional					Côte d’Ivoire			7–15		17	PF (aerobic fitness)				7
Dapi [67]	2011					Cross sectional					Cameroon			12–16		227	PA (self-report)				8
Puckree [68]	2011					Cross sectional					South Africa			10–12		120	PA (self-report) & SB (self-report)				7
Armstrong [69] ^c^	2011					Cross sectional					South Africa			6–13		10,295	PF (aerobic fitness, anaerobic fitness, musculoskeletal fitness, balance/flexibility)				10	
Adamo [70]	2011					Cross sectional					Kenya			9–13		179	PF (aerobic fitness, anaerobic fitness, musculoskeletal fitness)				7	
Naidoo [71]	2012					Cross sectional					South Africa			7–10		170	PF (aerobic fitness)				7	
Musa [72]	2012					Cross sectional					Nigeria			9–15		3,243	PF (aerobic fitness)				7	
Monyeki [73]	2012					Longitudinal					South Africa			14		256	PF (anaerobic fitness, musculoskeletal fitness)				8	
Onywera [74]	2012					Cross sectional					Kenya			9–12		169	PA (direct measures & self- report) & SB (self-report)				7	
Ojiambo [75]	2012					Cross sectional					Kenya			12–16		200	PA (direct measures & self -report) & SB (direct measures & self-report)				7	
Richards [76]	2012					Cross sectional					Uganda			11–14		31	PA (direct measures)				8	
Micklesfield [77]	2012					Cross sectional					South Africa			11–15		381	PA (self-report) & SB (self -report)				6	
Monyeki [78]	2012					Cross sectional					South Africa			14		256	PF (anaerobic fitness, musculoskeletal fitness)				8	
Monyeki [79]	2012					Cross sectional					South Africa			14–15		153	PA (self-report) & PF (aerobic fitness)				8	
Truter [80]	2012					Cross sectional					South Africa			9–13		280	PF (anaerobic fitness)				7	
Bovet [81] ^a,b^	2012					Cross sectional					Seychelles			9–16		8,462	PA (self-report)				9	
Pienaar [82]	2012					Longitudinal					South Africa			13–17		87	PA (self-report)				8	
Toriola [83]	2012					Longitudinal					South Africa			14		283	PA (self-report) & SB (self- report) & PF (aerobic fitness, anaerobic fitness, musculoskeletal fitness, balance/flexibility)				8	
Craig [84]	2012					Secondary analysis					South Africa			7, 11, 15		89	PA (direct measures)				9	
Ojiambo [85]	2012					Cross sectional					Kenya			13–16		200	PA (direct measures) & SB (direct measures)				7	
Malete [86]	2013					Cross sectional					Botswana			13–16		756	PA (self-report) & SB (self- report)				7	
Onywera [87]	2013					Cross sectional					Kenya			9–13		179	PA (direct measures)				7	
Heroux [88]	2013					Cross sectional					Kenya			9–13		179	PF (musculoskeletal fitness)				7	
														Total (*n*)		77,515	Average D&B score				7.5	

Notes: D & B score (Downs & Black score) [18]; PA = physical activity, SB = sedentary behaviour, PF = physical fitness. **^a^** = Article indicated targeting a sample size representative of the population of interest (2 articles). **^b^** = Article indicated recruiting a sample size representative of the population of interest (2 articles). **^c^** = Article indicated that the sample size was nationally representative (3 articles).

Data quality assessment revealed that the average score for all included studies was 7.5 out of a possible 10, pointing to a fairly high quality of evidence of the individual articles. The earliest relevant record captured was published in 1967, with two articles published between 1960 and 1969, none between 1970 and 1979, four articles between 1980 and 1989, six articles between 1990 and 1999, 26 articles between 2000 and 2009, and 33 articles published between 2010 and June 2013. This substantial increase in the publishing rate may be representative of a growing interest in this field of research. There was also good representation of the four regions of SSA with 20 (28.2%) articles from East African countries, 30 (42.3%) from South African countries, 16 (22.5%) from West African countries, two (2.8%) from Central African countries, and three (4.2%) that were combined.

### 3.1. Physical Activity

As shown in Table 5, 36 studies comprising 62,188 participants, presented in 40 articles, examined physical activity outcome(s). Of the 36 studies, 26 (72.2%) studies [26,29,34,35,37,38,43,45,47,52,55,56,58,60,61,62,63,64,67,68,77,79,81,82,83,86] used solely subjective measurements of physical activity (e.g., self-report or interviewer administered questionnaires), and 10 (27.8%) studies [30,31,32,33,42,53,65,74,75,76,84] used direct/objective measures (e.g., accelerometers, pedometers) or a combination of direct and subjective measures. Table 5 indicates the type the type of measure used and the main findings for each of the 36 studies. Despite the heterogeneity in the types of physical activity measures used in the included studies, some findings stood out from the data taken together.

**Table 5:** Findings from studies reporting on physical activity outcomes.

First Author [reference]	Year	Country	Age (years)				(n)			(n)					(n)				Main Finding(s) *
							Total			Male					Female				
Subjective Measures (Self-report or interviewer administered questionnaires)																			
Proctor [26]	1996	Cameroon	9–14				119			65					54				Physical activity among rural children was more than twice that of urban children, and was mostly work related and heavier intensity.
Prista [29]	1998	Mozambique	8–15				593			277					316				Underprivileged children had higher levels of physical activity due to survival activities (e.g., walking).
Prista [34]	2003	Mozambique	6–17				2,316			1,094					1,222				Low SES children and adolescents had higher levels of physical activity due to higher demands of survival activities and playing, but spent less time in formal sports than their more privileged peers.
McVeigh [35]	2004	South Africa	9				386			202					184				Inverted bell shape curve for association between percent physical activity levels and SES. The highest SES had the highest MVPA. Also discusses differences between different ethnicities.
Micklesfield [37]	2004	South Africa	7–11				198			0					198				Discussed differences between different ethnicities.
Subjective Measures (Self-report or interviewer administered questionnaires)																			
Larsen [38]	2004	Kenya				15–17					11				11			0	Village boys were significantly more habitually active (e.g., running, field work) than town boys.
Aandstad [43]	2006	Tanzania				9–10					156				87			69	88% of rural children walked to school, 71% for more than 15 min, and 34% participated in outdoor games after school on most or all days.
Djarova [45]	2006	Zimbabwe				6–14					49								Mean duration of activities during the day (hours): Sleeping 8.2, walking 2.4, sitting 3.9, standing 2.0, running 1.0, sports 2.5, playing 4.0.
Micklesfield [47]	2007	South Africa				9					64				36			28	Mean hours of participation in school physical education 1.1, school sports 1.4, informal activities 7.7, and weekend activities 8.
Lennox [52]	2008	South Africa				15					318				137			181	Lower SES school had higher activity levels, particularly, walking to school.
Peltzer [55]	2009	Namibia, Kenya, Uganda, Zimbabwe				13–15					12,740				7,517			5,223	Physical activity on ≥3 days (for ≥ 60 min) ranged from 24.4% to 36.0%. Active transport on ≥5 days (for ≥60 min) ranged from 19.8% to 31.1%.
Peltzer [56]	2010	Botswana, Kenya, Namibia, Senegal, Swaziland, Uganda, Zambia, and Zimbabwe				13–15					24,593				10,575			14,018	A range of 9.0% to 17.7% of children reported being physically active on ≥5 days (for ≥60 min). The average was 14.2% in the total sample, 16.6% in males, and 12.0% in females. 18.9% of the total sample was physically active on ≥3 days (for ≥60 min).
Subjective Measures (Self-report or interviewer administered questionnaires)																			
Senbanjo [58]	2010	Nigeria	5–14		392				202					190		Frequency of leisure time physical activity on ≥3 days in 5–9 year old males (3.2%), females (6.0%), in 10–14 year old males (20.1%), females (12.3%). Leisure time physical activity was higher in older children, in males, and in children of mothers with higher education level.			
Odunaiya [60]	2010	Nigeria	14–16		608											64.1% of the total sample participated in moderate and high physical activity.			
Ansa [61]	2010	Nigeria	10–17		964				472					492		75% of the total sample participated in outdoor sports. No SES effect found (not significant).			
Adeniyi [62]	2011	Nigeria	13–17		1,100				538					562		Females had higher risk of low physical activity than boys.			
Peltzer [63]	2011	Ghana & Uganda	13–15		5,613				2,738					2,875		78.5% of males and 84.9% of females were physically active for less than 60 min per day on at least 5 days per week.			
Naude [64]	2011	South Africa	12–16		162											33% of the total sample participated in organized sports.			
Dapi [67]	2011	Cameroon	12–16		227				108					119		Boys had significantly higher levels of physical activity than girls. No SES effect found (not significant).			
Puckree [68]	2011	South Africa	10–12		120				48					72		92% of the total sample participated in extracurricular sporting activities.			
Micklesfield [77]	2012	South Africa	11–15		381											Informal activity was significantly lower in older children. MVPA was significantly lower in girls than boys at all ages. Low SES was associated with higher walking for transport and lower MVPA in schools or clubs.			
Monyeki [79]	2012	South Africa	14–15		153				0					153		26.8% of girls participated in moderate and high physical activity.			
Bovet [81]	2012	Seychelles	9–16		8,462				4,239					4,223		There was higher leisure time physical activity in private compared to public schools, but higher active transport (walking) in public compared to private schools.			
Subjective Measures (Self-report or interviewer administered questionnaires)																			
Pienaar [82]	2012	South Africa	13–17	87				28					59				There was increased leisure time physical activity over time, higher house-chore related physical activity on weekends compared to weekdays, and higher exercise related activity on week days compared to weekends.		
Toriola [83]	2012	South Africa	14	283				111					172				Boys had higher physical activity in the high/vigorous level compared to girls.		
Malete [86]	2013	Botswana	13–16	756				464					292				Low SES and rural children had more minutes of physical activity than high SES or urban children.		
Direct/Objective Measures																			
Benefice [30]	1999	Senegal	13	221				0				221					Instrument: CSA accelerometer. Findings: Percentage of time spent during 12 hours of a day in MVPA (>940 counts/min corresponding to >6 METs) was 21.7% in the dry season, 29.3% in the rainy season, and 24.3% (corresponding to 2.9 h) in total.		
Benefice [31]	2001	Senegal	13	120 (40 at each visit)													Instrument: CSA accelerometer. Findings: Time spent in moderate and vigorous activity was 29.12% (corresponding to 4.4 h) at the 1st visit in 1997, 25.71% (corresponding to 3.9 h) at the 2nd visit in 1998, and 25.54% (corresponding to 3.8 h) at the 3rd visit in 1999. Daily physical activity was expressed as a physical activity level unit (ratio of energy expenditure to BMR). Three units of BMR corresponded to 890 counts/min. MVPA was therefore a BMR corresponding to >1,890 counts/min and was calculated as a percent of 15 h or time between 7 a.m. and 10 p.m.		
Garnier [33]	2001	Senegal	13–15	80				0				80					Instrument: CSA accelerometer. Findings: Migrants to the city spent more time engaged in moderate to heavy activity than did non-migrants in the rural area (9.3 h/24 h *vs*. 6.1 h/24 h). Intensity thresholds were defined according to Benefice & Cames, whereby MVPA was >610 counts/min or ≥3 METs [30].		
Direct/Objective Measures																			
Benefice [42]	2005	Senegal	10–13	99				46				53					Instrument: Cardio-frequencemeters. Findings: In adolescent girls, body composition was a significant predictor of activity levels.		
Prista [53]	2009	Mozambique	6–16	256				139				117					Instrument(s): Actigraph accelerometer and self-report measures. Findings: Software converted actigraph counts into relative energy exposure using the regression equation developed by Freedson *et al.* for children 6–18 years of age [90]. The average minutes of time spent in MVPA (≥3 METs) in boys and girls 6–8 years of age was 232.2 and 235.5, in boys and girls 9–11 years of age was 202.0 and 210.3, and in boys and girls 12–16 years of age was 157.4 and 158.7. This corresponds to range of 2.6–3.9 hours for the total sample.		
Croteau [65]	2011	Kenya	8–12	72				29				43					Instrument(s): Pedometers (step count data) and self-report measures. Findings: Daily steps in total sample 14,558 ± 3,993. Boys (16,262 ± 4,698) were significantly more active than girls (13,463 ± 3,051). No significant effect for age was found. Observed daily steps are higher than those observed in the USA and samples in other developed countries.		
Onywera [74]	2012	Kenya	9–12	169				85				84					Instrument(s): Pedometers (step count data) and self-report measures. Findings: Rural children were more physically active than urban children. 87% rural children and 42% urban children used active transport to get to school.		
Ojiambo [75]	2012	Kenya	12–16	200				99				101					Instrument: Actigraph accelerometer. Physical activity levels were assessed using cut-points developed by Puyau *et al.* whereby MVPA >3,200 counts/min [91]. The mean minutes of MVPA were 54 (corresponding to 0.9 h) in the total sample, 68 in rural males, 62 in rural females, 50 in urban males, and 37 in urban females.		
Direct/Objective Measures																			
Richards [76]	2012	Uganda	11–14	31				31					0				Instrument: Actigraph accelerometer. Findings: Mean minutes per day of MVPA were 114.09 (corresponding to 1.9 h), with only 4.32 minutes in the vigorous range. (MVPA cut-points not indicated).		
Craig [84]	2013	South Africa	7–15	89				46					43				Instrument: Actigraph accelerometer. Findings: Total physical activity was generally high (mean accelerometer counts per minute ranged 485–1017), but MVPA was low with less than 1% of the total sample meeting the MVPA guidelines using the Puyau *et al.* cut points [91]. MVPA in boys comprised 1.7% of waking hours (12 min/day) at age 7, 1.3% (10 min/day) at age 11, and 0.6% (4 min/day) at age 15. MVPA in girls comprised 1.5% (11 min/day) at age 7, 0.9% (6 min/day) at age 11, and 0.2% (1 min/day) at age 15 years.		

Notes: SES (socioeconomic status); MVPA (moderate-to-vigorous physical activity); Counts/min (counts per min); BMR (basal metabolic rate); METs (metabolic equivalents). Table excludes references [32,36,85,87] which used an identical study sample as used in another included manuscript. * Main finding(s) from the article as relates to physical activity outcomes.

#### 3.1.1. Physical Activity Transition

Of the articles that used direct measures, seven studies [30,31,33,53,75,76,84] used accelerometry to measure physical activity. Only one study examined the changing trends over time. Benefice *et al.* found a decline in the amount of time spent in moderate-to-vigorous intensity physical activity (MVPA) from 29.12% (corresponding to 4.4 h) in 1997, to 25.71% (3.9 h) in 1998 and 25.54% (3.8 h) in 1999 [31]. Studies that used accelerometry used varying MVPA cut-points to categorise their samples making it impossible to comment on trends over time. As such, the general trend towards decreasing proportions of MVPA from earlier studies (1999–2009) with sample means of ≥2.6 h of MVPA [30,31,33,53] to later studies (2012) with sample means of ≤1.9 h of MVPA [75,76,84] as observed in Table 5*,* may be related to more stringent cut-points rather than decreasing levels of physical activity in these samples. In general, a lack of population-representative samples and heterogeneity of measurement protocols did not allow for a clear assessment of physical activity level trends over time. 

One large scale, multi-country study using subjective measures with a sample of 24,593 participants reported that 14.2% (16.6% of males, 12.0% of females) of children were physically active on 5 days or more, for at least 60 min/day during leisure time [56]. The proportion was 18.9% of children on 3 days or more, for at least 60 min/day during leisure time [56]. Step count data determined that the observed average number of daily steps was higher than those of the United States and samples in other developed countries [65].

#### 3.1.2. Socioeconomic (SES) and Urban/Rural Differences

Of the 14 studies using subjective measures to examine the association between physical activity and SES, 11 found that lower SES and rural children had higher levels of physical activity compared to higher SES and urban children, or children who had mothers with a higher educational level [26,29,34,38,43,52,58,74,77,81,86], while three found contradicting evidence or no SES differences [35,61,67]. Lower SES and rural children engaged in higher levels of active transportation (e.g., walking and running to school) [43,52,74,77,81], spent more time in activities of daily living (e.g., house chores, work related, habitual activity) [26,29,34,38], but, spent less time engaged in organized sports or formal activities compared to their higher SES and urban peers [77,81]. Directly measured physical activity (both accelerometry and pedometer data) also found that rural children were more physically active than urban children [74,75]. 

#### 3.1.3. Sex Differences

Of the six studies using subjective data collection methods and that examined the differences in physical activity trends in boys and girls, five studies found higher levels of physical activity (e.g., informal activity, leisure time physical activity, MVPA) in boys compared to girls irrespective of age [56,63,67,77,83], while one study found that this was only true in the higher age-group (10–14 years), but not in lower age-group (5–9 years) [58]. Similarly, directly measured physical activity (both accelerometry and pedometer data) found that boys were significantly more active than girls [65,84].

### 3.2. Sedentary Behaviours

As shown in Table 6, a total of 17 studies examining sedentary behaviours, comprising 33,525 participants, were included in this review. Of these, the majority (12 studies) used self-report measures [35,47,52,55,56,63,64,68,74,77,83,86], and five studies used direct measurements [33,39,44,75,85]. Specific measurement tools varied across studies, but total weekday and/or weekend television viewing time was most often studied. All studies examining sedentary behaviour were published after 2000, with the majority published in the last three years (*i.e.*, since 2010). 

**Table 6:** Findings from studies reporting on sedentary behaviour outcomes.

First Author [reference]	Year		Country			Age (Years)					(n) Total		(n) Male			(n) Female			Main Finding(s) *
Subjective Measures (Self-report or interviewer administered questionnaires)																			
McVeigh [35]	2004		South Africa			9					386		202			184			Sedentary activity (h/day) among white girls 8.61 (±0.54), black girls 9.03 (±0.34), white boys 10.48 (±0.79), and black boys 10.63 (±0.33).
Micklesfield [47]	2007		South Africa			9					64		36			28			Average min per day spent on television watching was 123 ± 8.3.
Lennox [52]	2008		South Africa			15					318		137			181			TV h during the week in school 1 “(lower SES) & school 2 (higher SES) among boys 1.92 (1.43) & 2.24 (1.27), in school 1 & 2 among girls 1.76 (1.32) & 2.53 (1.38). TV hrs during the weekend in school 1 & 2 among boys 3.31 (2.10) & 3.62 (2.50), in school 1 & 2 among girls 2.74 (1.94) & 3.28 (2.38).
Peltzer [55]	2009		Namibia, Kenya, Uganda, Zimbabwe			13–15					12,740		7,517			5,223			Proportion of children reporting less than 3 h of sitting/day was 56.6% for Kenya, 64.3% for Namibia, 70.3% for Uganda, and 56.3% for Zimbabwe.
Peltzer [56]	2010		Botswana, Kenya, Namibia, Senegal, Swaziland, Uganda, Zambia, and Zimbabwe			13–15					24,593		10,575			14,018			Overall time spent sitting on a usual day for <1 h was 39.4%, 1–2 h was 32.7%, 3–4 h was 17.5%, ≥5 h was 11.2%.
Peltzer [63]	2011		Ghana and Uganda			13–15					5,613		2,738			2,875			Sedentary behavior (≥ 3h/day) in males was 27.1% (23.6–30.5), in females was 26.9% (24.3–29.6).
Naude [64]	2011		South Africa			12–16					162								Mean total weekly time (min) spent watching TV or playing on the computer was 1,001 (±570).
Puckree [68]	2011		South Africa			10–12					120		48			72			All sampled children reported watching >4 h of TV on weekdays and 6–8 h on weekends.
Onywera [74]	2012		Kenya			9–12					169		85			84			30% of rural children spent >2 h/week on screen time compared with 50% of urban children, and 62.5% of the rural children spent 0 h/week playing screen games compared with 13.1% of urban children.
Micklesfield [77]	2012		South Africa			11–15					381								Sedentary activity was significantly higher in older girls compared to younger counterparts (*p* < 0.05). Increasing pubertal status was associated with an increase in sedentary activity. Lower SES at the maternal, household, and community level was significantly associated with less sedentary activity.	
Toriola [83]	2012		South Africa			14					283		111			172			Overall, 18%, 23%, and 40% watched TV for >3 h, 2–3 h, <1 h per day, respectively. More girls (19%) than boys (16%) watched TV for >3 h/day.	
Malete [86]	2013		Botswana			13–16					756		464			292			Time spent sitting (min/week) was 2,612.38 (±910.87) or 6.2 h/day.	
Direct/Objective Measures																			
Garnier [33]	2001		Senegal			13–15					80		0			80			Instrument: CSA accelerometer. Findings: Sleeping and inactivity of migrants (who move for work) was 6 h and 42 min, and of non-migrants 8h and 29 min.	
Benefice [39]	2004		Senegal			13–15					40 (in each year)		0			40 (in each year)			Instrument: CSA accelerometer. Findings: Resting time (min)—defined as the number of zero counts during the day—was 93 (±54) in the year 1997, 111 (±65) in 1998, and 103 (±51) in 1999.	
Garnier [44]	2006		Senegal			13–15					80		40			40			Instrument: CSA accelerometer. Findings: Girls were more inactive (4 h and 23 min) than boys 2 h and 49 min).	
Ojiambo [75]	2012		Kenya			12–16					200		99			101			Instrument: Actigraph accelerometer. Findings: Min (%) sedentary time in total sample was 584 ± 113 (72%); in rural males was 555 ± 67 (65%), in rural females was 539 ± 91 (66%), in urban males was 678 ± 95 (78%), and in urban females was 694 ± 81 (80%).	
Ojiambo [85]	2012		Kenya			13–16					200		98			102			Instrument: Actigraph accelerometer. Findings: Daily mean sedentary time (min) was 619 ± 109, or approximately 78% of the monitored time.	

Notes: SES (socioeconomic status); TV (television). * Main finding(s) from the article as relates to sedentary behaviour outcomes.

#### 3.2.1. Sedentary Behaviour Transition

In South Africa, self-reported time spent watching television was inconsistent. Recent work by Puckree *et al.* showed that 100% of participants aged 10–12 years watched more than 4 h of television on weekdays, and 6–8 h of television on weekends [68]. Toriola and Monyeki reported that 40% of 14 year old participants watched less than 1 hour of television per day [83]. In other studies, the self-reported mean television viewing time was between 2 and 2.5 h per day for 9–16 year old children, with weekend television viewing slightly higher at 2.7 to 3.6 h, and television viewing generally higher in older children [47,52,64]. Data from Ghana and Uganda found that 27% of children aged 13–15 years spent more than 3 h per day in sedentary pursuits [63], while in Botswana, 14 year olds spent an average of 6.2 hours per day sitting [86]. Senegalese youth reported only 1.33 to 1.41 h of sedentary time per day [39].

One multi-country comparative study found that in children 13–15 years of age, the proportion reporting less than 3 h of sitting per day was 29.7% in Uganda, 25.7% in Namibia, 43.4% in Kenya, and 43.7% in Zimbabwe [55]. Another large scale multi-country study using data from eight African countries (Botswana, Kenya, Namibia, Senegal, Swaziland, Uganda, Zambia, and Zimbabwe) found that 39.4% of children aged 13–15 years spent less than one hour per day sitting, and an additional 32.7% spent 1–2 h per day sitting when not in school or doing homework [56].

Notably, the short time span within which the studies reporting on sedentary behaviour were published made it impossible to examine transitions over time, since temporal trends are often reasonably stable over short periods of time.

#### 3.2.2. Sex Differences

Accelerometry measured sedentary time showed that Senegalese girls spent more time in sedentary behaviours than boys (4.23 h *vs*. 2.49 h) [44]. Investigation of self-reported television viewing also found that more girls (19%) than boys (16%) watched television for more than 3 hours daily [83]. Most studies however either found no significant difference or did not report on the difference in sedentary behaviours between boys and girls [35,52,63,75]. 

#### 3.2.3. Age Differences

One included study examined age differences and found that sedentary activity was significantly higher in older girls compared to their younger counterparts [77]. This study also showed that increasing pubertal status, which is not a proxy for age, was associated with an increase in sedentary behaviours, but that the most significant determinant of sedentary behaviour was SES [77].

#### 3.2.4. SES and Urban/Rural Differences

Higher SES and urban living children in Kenya were found to spend significantly more time in sedentary pursuits than their lower SES and rural counterparts, with approximately 50% of the urban children, and only 30% of the rural children reporting spending over 2 hours each week on screen time activities [74,75]. This is consistent with the findings of Lennox *et al.* showing that South African children in higher SES schools spent more time watching television than children in lower SES schools on both weekdays and weekends [52]. Additionally, Micklesfield *et al.* found that higher maternal, household, and community level SES were significantly associated with increased sedentary time [77]. In contrast, Malete *et al.*, found that public school students and those living in rural villages (lower SES) reported significantly more minutes of sitting than students in private schools or students from cities (higher SES); which is an unexpected finding given that the former group also reported more vigorous and total physical activity than the latter [86]. 

### 3.3. Physical Fitness

Thirty six included studies, presented in 37 papers, comprising 30,452 participants, examined physical fitness measures. The majority of these studies were published in the past three years, but studies were included from as early as 1967 [19]. Almost half (47.2%) of the 36 fitness related studies included South African participants. All studies used direct measures of physical fitness. Studies are presented based on whether they examined aerobic fitness, anaerobic fitness, musculoskeletal fitness and strength, or balance and flexibility, with many of the studies examining more than one aspect of physical fitness. 

#### 3.3.1. Aerobic Fitness

Twenty one studies presented data on aerobic fitness. Aerobic fitness was measured through shuttle run [29,41,43,48,49,51,52,57,66,69,70,71,72,79,83,88], maximal cycle ergometer test [20,54], maximal aerobic capacity [52,59], distance run [23,29,41,48,51], and the Harvard Step test [21]. Boys were found to have higher aerobic capacity and performed considerably better on measures of aerobic fitness than girls [23,41,49,54,59,66,72,83,88].

Two studies found that lower SES and rural children performed better on measures of aerobic fitness than higher SES and urban children [52,70]. In contrast, one study found the opposite to be true when comparing girls in the townships (lower SES) to girls in the towns (higher SES) [79]. It is noteworthy that in the latter study, township girls were also found to have significantly higher prevalence of underweight than the town girls. Aandstad *et al.* found that Tanzanian children had better aerobic fitness test results when compared to a Norwegian reference group [43]. 

Only one study examined changes over time in aerobic fitness tests. In their study of 702 rural South African children, Monyeki *et al.* found significant improvements in aerobic fitness results (shuttle run and 1,600 m run tests) from 2001 to 2002 though this was only across one year [48]. However, when data from studies using similar measures of aerobic fitness (*i.e.*, shuttle run [41,48,51,69] and 1,600 m run [41,48,51]) were examined, we found largely unaltered trends in aerobic fitness over time, with boys performing better than girls as shown in Table 7. 

#### 3.3.2. Anaerobic Fitness

Seventeen studies presented data on anaerobic fitness. This was measured though timed dashes/sprints [19,25,27,28,40,41,48,49,50,70] or long jumps [25,27,28,40,41,48,49,50,51,69,73,78,83]. Boys performed better in all measures of anaerobic fitness than girls [41,49,83]. Anaerobic test parameters in Senegalese children were found to be inferior to those of well-nourished Western children [25,28]. No temporal trend evidence was available.

**Table 7:** Findings from studies reporting on comparable physical fitness outcomes.

First Author [reference]	Year			Country		Age (Years)		(n)		(n)			(10 × 5m) Shuttle Run (sec)				Run 1,600 (m)				Grip Strength (kg)			Sit & Reach (cm)	
								Boys		Girls															
													B		G		B		G		B		G	B	G
Corlett [24]	1988			Botswana		7 (Urb)		18		17											11.9		11.6		
																					(0.3)		(1.0)		
						7 (Rur)		19		31											7.4		6.3		
																					(1.3)		(1.1)		
						8 (Urb)		26		29											12.4		11.6		
																					(0.8)		(0.5)		
						8 (Rur)		25		24											9.2		7.8		
																					(1.1)		(1.6)		
						9 (Urb)		18		30											13.4		12.7		
																					(0.9)		(0.8)		
						9 (Rur)		32		37											9.8		8.4		
																					(1.1)		(1.0)		
						10 (Urb)		28		20											15.6		14.0		
																					(1.2)		(0.6)		
						10 (Rur)		43		25											12.4		10.0		
																					(2.2)		(1.7)		
						11 (Urb)		24		18											17.4		15.1		
																					(1.3)		(1.1)		
						11 (Rur)		30		38											13.7		12.8		
																					(1.7)		(2.0)		
						12 (Urb)		17		16											20.2		16.8		
																					(1.4)		(1.5)		
						12 (Rur)		36		32											16.5		15.2		
																					(2.3)		(2.8)		
Monyeki [48] *	2001			South Africa		7–10		152		133			22.9		23.2		489.8		531.2						
													(2.1)		(1.9)		(42.0)		(62.9)						
	2002					7–10		228		189			21.9		21.8		475.3		514.6						
													(2.1)		(2.1)		(48.0)		(57.7)						
	2001					11–15		152		133			22.0		22.2		452.9		511.8						
													(1.8)		(1.7)		(40.5)		(59.8)						
	2002					11–15		228		189			21.2		21.5		460.7		529.3						
													(1.9)		(1.6)		(53.8)		(79.6)						
Monyeki [40]	2004			South Africa		7		47		38														14.1	15.0
																								(4.1)	(5.1)
Monyeki 41]	2005			South Africa		7		46		36			24.6		24.3		516		546					14.1	14. 4
													(2.3)		(3.1)		(36.5)		(42.8)					(4.2)	(5.1)
						8		58		54			22.8		23.3		486		528					15.5	16.5
													(1.8)		(1.8)		(32.5)		(60.4)					(4.8)	(3.8)
						9		71		60			22.0		22.6		474		522					14.3	15.6
													(1.5)		(1.7)		(43.1)		(65.1)					(5.1)	(4.6)
						10		80		70			22.0		22.8		456		510					14.4	15.8
													(2.2)		(1.9)		(39.6)		(53.0)					(5.1)	(4.6)
						11		69		74			21.8		22.1		450		498					15.7	16.9
													(1.9)		(1.7)		(41.0)		(50.3)					(5.1)	(5.1)
						12		87		67			21.8		21.9		450		510					13.8	17.8
													(1.6)		(1.5)		(40.4)		(44.1)					(4.8)	(5.0)
						13		35		21			21.3		21.6		438		504					12.8	17.6
													(1.2)		(1.3)		(34.4)		(83.1)					(5.3)	(4.2)
						14		16		11			20.7		21.5		408		528					14.1	19.5
													(1.2)		(1.1)		(56.7)		(89.9)					(5.2)	(6.2)
Onyewadume [46]	2006			Botswana		11–14		15		15											34.6		24.1		
																					(7.3)		(5.1)		
Monyeki [51]	2008			South Africa		7		80		61			23.8		23.7		549.2		570.7						
													(2.6)		(2.9)		(94.6)		(69.0)						
						8		86		82			23.0		23.3		523.6		563.6						
													(2.3)		(2.6)		(76.3)		(87.2)						
						9		107		89			22.7		22.3		500.2		535.0						
													(2.2)		(2.0)		(78.9)		(80.9)						
						10		102		108			22.3		22.4		496.7		527.6						
													(1.8)		(1.7)		(84.0)		(67.6)						
						11		111		94			21.9		21.8		479.5		526.9						
													(2.1)		(1.8)		(64.3)		(74.2)						
						12		99		82			21.7		21.9		475.5		523.0						
													(1.7)		(1.9)		(83.1)		(82.2)						
						13		49		42			21.4		21.6		463.9		501.9						
													(1.4)		(1.3)		(96.9)		(128.6)						
Lennox [52]	2008			South Africa																				School 1	
						15 (RR)		116		136														29.0	31.1
						15 (RL)		116		136														29.1	31.5
																								School 2	
						15 (RR)		21		45														27.5	29.1
						15 (RL)		21		45														27.6	28.6
Armstrong [69]	2011			South Africa		6–13		5,611		4,684			22.8		23.8									21.4	23.7
Toriola [83]	2012			South Africa		14		111		172														42.2	48.5
																								(9.1)	(7.3)
Heroux [88]	2013			Kenya		9–13		86		93											34.7		31.1		
																					(32.0,37.3)		(28.7, 3.5)		

Notes: B (boys); G (girls); Urb (urban); Rur (rural); RR (reach-right); RL (reach-left). Where reported, variance included in brackets as (standard deviation) or (confidence intervals). School 1 had lower socioeconomic status than school 2 [52]. (*) Study was published in 2007, but data was collected in the years indicated, *i.e.*, 2001 and 2002.

#### 3.3.3. Musculoskeletal Fitness and Strength

Twenty five studies examined musculoskeletal fitness and strength. This was measured via bent arm hang [41,48,51,73,78,83], grip strength [20,24,25,27,29,45,46,47,50,52,68,70,88], timed push-ups [46,49], timed sit-ups [19,29,40,41,46,48,49,51,52,69,73,78,83], or throwing [19,25,27,28,49,69]. Boys performed better than girls in measures of bent arm, sit-ups, and push-ups [41,83].

As shown in Table 7, when data from studies using a similar measure of grip strength (kilograms) were examined, we found a trend towards increasing scores in both boys and girls over time [24,46,88]. It is however important to note that geographical differences between Botswana [24,46] and Kenya [88] may be clouding these findings. Further, even within the studies from Botswana, there was little overlap among the age groups, further complicating temporal trend analysis. From these data, it was also determined that boys performed better than girls in grip strength measurements [24,46,88]. Grip strength seemingly increased with age [50], and was found to be higher in higher SES and urban children compared to lower SES and rural children [24,70]. In general, measures of skeletal muscle fitness and strength were found to be lower in SSA children compared to various Western reference samples (e.g., United States, the Netherlands) [22,25,28,50].

#### 3.3.4. Balance and Flexibility

Eight studies presented data on balance and flexibility. This was measured via flamingo balance [41,51] and sit-and-reach test [22,40,41,50,52,69,83]. Girls were more flexible and performed better in balance tests than boys [41,50,83]. As shown in Table 7, when data from studies examining sit and reach (cm) performance were assessed, we found a trend towards increasing measures of flexibility in both sexes, with girls performing consistently better than boys [40,41,52,69,83]. It is however important to note that these apparent increases may be a function of the way in which sit-and reach performance was scored, since we observe an approximate 3-fold increase in scores over an 8 year period among South African children [40,41,52,69,83].

## 4. Discussion

This is the first systematic review to comprehensively explore the evidence for a physical activity and fitness transition, and their correlates, among SSA’s children and youth. 

### 4.1. Physical Activity Transition

Subjective methods of assessing physical activity are commonly used to measure types, frequencies, durations, and levels of physical activity. This is likely due to low costs associated with measurement [92]. Previous work however has shown that there is little association between self-report and directly measured physical activity; largely as a result of poor recall in children [92,93,94,95]. A majority of the included studies examining physical activity used subjective methods of data collection. A smaller proportion of studies used direct methods to assess physical activity, thereby providing higher accuracy and validity [96]. The means of measured MVPA reported in studies that used accelerometry to monitor physical activity ranged from 1 min/day to 9.3 h/day [30,31,33,53,75,76,84]. The only included study that examined changes in reported time spent in MVPA found a decline in MVPA of 30 minutes per day from 1997 to 1999 [31]. Regrettably, those using direct methods also used vastly different measurement devices and cut-points, making it impossible to conduct legitimate quantitative analyses of the results. 

Assessment of a physical activity time trend was challenging given that many of the subjective instruments used have yet to be properly validated and are fraught with various limitations, while the objective measures used were mainly cross-sectional and differed in methodology [97]. Owing to the vast heterogeneity of these results, little could be said definitively on whether there was indeed evidence of a physical activity transition over time. It is imperative that future work examine the duration and intensity of physical activity on population representative samples using common measurement techniques and sampling procedures. 

Similarly, a clear transition to increasing time spent in sedentary behaviours was not apparent. The majority of studies reporting on sedentary behaviour outcomes were published after 2010, further complicating analysis of trends over time. The reported means of time spent in sedentary pursuits ranged from 1.3 hours to 6 hours on weekdays, and were as high as 8 h on weekends [39,47,52,64,68,86]. These means are problematic given that the Canadian sedentary behaviour guidelines (the first published evidence-based guidelines on sedentary behaviour) recommend that children 5–17 years should limit their recreational sedentary screen time to no more than 2 h per day [14]. The urbanization trend suggests an increase in sedentary behaviours over time as the data revealed higher television viewing times among urban and higher SES children than rural and lower SES children.

Quantitative and narrative analyses of studies examining physical fitness measures revealed a tendency towards increased fitness measures, particularly in grip strength and flexibility over time in SSA, though the inconsistencies in measures used and representativeness of the samples makes direct comparisons across studies tenuous. It has been postulated that body composition indices should be interpreted differently in undernourished populations, that is, higher body mass index measures could be seen as a measure of muscle mass instead of a measure of fatness [41,48]. With this in mind, whereas previously the prevalence of underweight in SSA’s school-aged children was high, these numbers are falling, giving rise to higher proportions of well-nourished children who may have greater muscle mass and perform better in various fitness measures. Improvements in fitness parameters in SSA’s school-aged children may also be as a result of an increase in the proportion of children participating in formal/organized sports or activities, which has enabled these children to improve certain physical fitness skills that may not be learned through informal activities, random play, and self-directed activities engaged in more by the rural living and lower SES children. Despite these apparent improvements, when compared to Western reference groups, SSA children performed better in aerobic fitness measures but worse in anaerobic fitness tests or measures of musculoskeletal fitness and strength [22,25,28,43].

### 4.2. SES Differences

This systematic review found that lower SES and rural living children had higher levels of physical activity compared to higher SES and urban living children. Higher activity levels in lower SES children are associated with higher demands of informal/survival activities such as household chores and walking from place to place (active transport), rather than the formal/organized sporting activities engaged in by their more privileged peers. Higher SES and urban living children were also found to spend more time in sedentary pursuits than lower SES and rural living children; consistent with the finding that lower SES children were more active. This finding is likely due to lower access to motorized transport and higher need for their contribution to household and other tasks in lower SES settings. Lastly, lower SES children were also found to perform better in aerobic fitness measures compared to their higher SES peers. Superior aerobic fitness performance in lower SES and rural children may indeed be as a direct result of their higher participation in habitual active transport (e.g., walking, running), when compared to higher SES and urban children. In the one study where lower SES girls performed worse than their higher SES peers, this may be explained by the high prevalence of underweight that was also found in the low SES group, resulting in lower muscle mass as previously described; hence, poor performance in aerobic fitness tests [41,48,79]. The same may be said about the finding that higher SES children perform better in grip strength measures than lower SES children. 

### 4.3. Sex Differences

This review found significantly higher levels of physical activity in boys compared to girls irrespective of age. Similarly, boys engaged in less sedentary behaviours (e.g., television viewing) than girls. Boys were also found to have considerably better aerobic, anaerobic, musculoskeletal fitness, and strength measures than girls; however, girls performed better on balance and flexibility measures of fitness. Besides biology and socio-cultural roles, this superior physical and functional ability of boys may be explained by their higher motivation to participate in physical activities. 

The use of high quality methodology to capture and synthesize the studies in this review offers strength to the findings and conclusions in this manuscript. Additionally, decisions were made *a priori* to limit possible bias, and review processes were conducted in duplicate to ensure a higher level of accuracy. However, this review had several limitations including the vast heterogeneity in study sampling and methodology, which complicated quantitative analysis and direct comparison. Further, any trends observed over time may be reflective of the growth or differences in the ages of participants rather than group differences. It is also unclear if any material relevant for this review may have been published in un-indexed journals and hence not captured by the literature search. 

## 5. Concluding Remarks

Owing to the vast heterogeneity in measurement devices, methodologies, and cut-points used by studies included in this review, little could be said definitively on the evidence for a physical activity and fitness transition over time. More generally however, the data revealed that urbanization was associated with a developing trend towards decreasing physical activity, increasing sedentary behaviours, and decreasing fitness measures (particularly aerobic fitness) over time. This was shown by engagement in lower amounts and levels of physical activity, higher television viewing and other sedentary pursuits, and lower aerobic fitness levels among urban and higher SES children compared to their rural and lower SES peers. As such, proactive strategies to prevent decreased physical activity and fitness and increased sedentary behaviours in the context of a probable physical activity transition in children from SSA appears warranted. 

This systematic review also revealed a critical lack of representative, temporally sequenced data on physical activity, sedentary behaviours, and physical fitness measures in SSA’s school-aged children and youth, which is a largely understudied and vulnerable group [98]. It is our recommendation that future work entail concerted efforts in carrying out nationally representative surveys, using comparable or common measurement techniques, sampling procedures, and cut-points, in order to effectively monitor physical activity transitions over time in this region. Further, considering that costs and ease of accelerometry use have improved over the last decade, we recommend that objective monitoring of physical activity and sedentary time be used in future studies. For instance, multi-country surveys would best strengthen the knowledge base in this field of research. 
